# Qualitative Distinction of Autotrophic and Heterotrophic Processes at the Leaf Level by Means of Triple Stable Isotope (C–O–H) Patterns

**DOI:** 10.3389/fpls.2015.01008

**Published:** 2015-11-24

**Authors:** Adam Kimak, Zoltan Kern, Markus Leuenberger

**Affiliations:** ^1^Climate and Environmental Physics, Physics Institute, University of BernBern, Switzerland; ^2^Oeschger Centre for Climate Change Research, University of BernBern, Switzerland; ^3^Institute for Geological and Geochemical Research, Research Centre for Astronomy and Earth Sciences, Hungarian Academy of Sciences (MTA)Budapest, Hungary

**Keywords:** leaf cellulose, hydrogen, oxygen, carbon, isotope, seasonal, intra-annual, juvenile-mature foliage

## Abstract

Foliar samples were harvested from two oaks, a beech, and a yew at the same site in order to trace the development of the leaves over an entire vegetation season. Cellulose yield and stable isotopic compositions (δ^13^C, δ^18^O, and δD) were analyzed on leaf cellulose. All parameters unequivocally define a juvenile and a mature period in the foliar expansion of each species. The accompanying shifts of the δ^13^C-values are in agreement with the transition from remobilized carbohydrates (juvenile period), to current photosynthates (mature phase). While the opponent seasonal trends of δ^18^O of blade and vein cellulose are in perfect agreement with the state-of-art mechanistic understanding, the lack of this discrepancy for δD, documented for the first time, is unexpected. For example, the offset range of 18 permil (oak veins) to 57 permil (oak blades) in δD may represent a process driven shift from autotrophic to heterotrophic processes. The shared pattern between blade and vein found for both oak and beech suggests an overwhelming metabolic isotope effect on δD that might be accompanied by proton transfer linked to the Calvin-cycle. These results provide strong evidence that hydrogen and oxygen are under different biochemical controls even at the leaf level.

## Introduction

Stable isotopes in plant physiology are used across a broad scale. They have also been applied to paleoclimate studies since tree-ring carbohydrates act as fingerprints of current climatic conditions (Gessler et al., 2014; Saurer et al., 2014). In previous decades, most of the studies were performed using only a single isotopic composition rather than a multi-isotope approach (Scheidegger et al., 2000). The following can be summarized for each element and its isotopic composition.

### Carbon

Carbon isotopes are assimilated from atmospheric carbon-dioxide, carrying intra-annual variations of δ^13^C of carbon-dioxide (CO_2_) (Keeling et al., 1995). According to Farquhar et al. (1982), photosynthesis discrimination against ^13^C by Rubisco is dependent on the ratio of intercellular to atmospheric CO_2_ concentrations (c_i_/c_a_), which are themselves controlled by stomatal conductance and driven by the CO_2_ assimilation rate (Farquhar et al., 1989). However, annual tree ring formation is partly supported by stored carbohydrates, especially at the beginning of the growing season, thus inducing higher degrees of coherence with previous years (Helle and Schleser, 2004; Kagawa et al., 2006; Cernusak et al., 2009). Furthermore, similar seasonal variability of δ^13^C has been also found in ryegrass. However, an opposite trend has been documented for δ^15^N (Wang and Schjoerring, 2012). Since, carbon isotopes originate from atmospheric CO_2_ driven by climate associated variables, δ^13^C measurements of tree rings are frequently used for the determination of past weather extremes (Raffalli-Delerce et al., 2004; Kress et al., 2010), and changes in past water use efficiency (Saurer et al., 2014) among others. However, these regional signals stored in tree rings can be altered or concealed by local conditions (Saurer et al., 1997).

### Oxygen

Oxygen (and hydrogen) isotopes originate from source water, taken up mainly as groundwater, although in some special cases fog, dew, or water vapor in general can also act as alternative water sources (Roden et al., 2009; Studer et al., 2015).

According to the depths of water uptake, the source water can be fractionated by evaporative gradients in the soil, in particular for shallow-rooting trees, providing possible seasonal variations of δ^18^O as well as significant phase-shifts (White, 1989; Treydte et al., 2014) in source water (Ehleringer and Dawson, 1992; Gessler et al., 2014). In addition, no fractionation has been observed from roots to leaves when the water is transported from soil into the xylem of roots, trunk and twigs (White et al., 1985; Dawson and Ehleringer, 1993; Treydte et al., 2014).

Further processes do influence leaf water oxygen isotopes, such as evaporative enrichment during transpiration (Farquhar and Lloyd, 1993) and the Péclet-effect, which represents the modified Craig-Gordon model by including the correction of the diffusion of evaporative ^18^O-enriched water back to the leaf lamina by advective transport of unenriched xylem water (Farquhar and Lloyd, 1993; Gessler et al., 2014). Moreover, almost every metabolic step is followed by a certain degree of isotope fractionation, resulting in an overall enrichment of plant organic matter by +27‰ compared to lamina leaf water (Deniro and Epstein, 1981; Yakir and Deniro, 1990).

### Hydrogen

At the leaf level water molecules are split in photosynthesis. Hydrogen from leaf water is transferred to nicotinamide adenine dinucleotide (NADP^+^), which is further transformed to NADPH which in turn strongly prefers light hydrogen (^1^H), resulting in a very depleted isotopic composition for hydrogen (Yakir and Deniro, 1990; Luo et al., 1991). However, further metabolic steps, such as the Calvin-cycle (Yakir, 1992; Hayes, 2001), the exchange of C-bound H with cell water (Luo and Sternberg, 1992; Yakir, 1992) and the biosynthesis of plant organic material (Yakir and Deniro, 1990; Luo and Sternberg, 1992) initiate isotopic enrichments in hydrogen isotopic composition.

Although, the isotopic fractionations of metabolic processes, including carbon- (Gessler et al., 2008), oxygen- (Barbour et al., 2004), and hydrogen-isotopes (Roden and Ehleringer, 1999), are mostly well-understood (Gessler et al., 2014), a complete seasonal variability of isotopic composition (C–O–H) in leaf cellulose, containing all the major constructive elements has yet to be presented.

The development of leaves, similar to the case with tree rings, is influenced by previous years' stored carbohydrates and current climate conditions, and is driven by species-specific turnover from heterotrophic- to autotrophic-based metabolism (Keel et al., 2007; Keel and Schädel, 2010). At the beginning of the growing season, the major impact on cellulose synthesis is generated by remobilized carbohydrates traced by tree ring (Helle and Schleser, 2004; Kagawa et al., 2006) and leaf cellulose analyses (Leavitt and Long, 1985; Stokes et al., 2009). However, the inter- and intra-annual distribution of carbohydrate storage/usage can vary, depending on local climate conditions (Kimak and Leuenberger, 2015). In addition, the remaining soluble carbohydrates that support the tree against frost damage in winter, described by the increasing soluble carbohydrate concentration in the xylem of roots, stem, and branches (Loescher et al., 1990; Barbaroux and Bréda, 2002; Barbaroux et al., 2003; Helle and Schleser, 2004), can be also used for plant organic matter formation in spring. According to Eglin et al. (2009), the aforementioned effect of remaining carbohydrates was also documented at leaf level, including leaf cellulose, starch, and water-soluble fractions. After the first leaf cells are developed and are capable of photosynthesis, the usage of reserves decreases and concurrently the usage of newly assimilated carbohydrates increases until it becomes the major source of cellulose synthesis. The period during which the tree uses carbohydrate from reserves in parallel with direct assimilates for cellulose formation is called earlywood (EW) (Huang et al., 2014). In deciduous species, the earlywood cells contain typically large vessels (in particular in oaks) for the increased nutrient and water transport at the beginning of growing season. The period of the growing season dominated by autotrophic metabolism is called latewood (LW), representing smaller cells besides the higher cell density and excluding the aforementioned large vessels (Feuillat et al., 1997). Consequently, newly assimilated carbohydrates used for LW cells record the current climate conditions. This is why LW is generally used for climate research in preference to EW (McCarroll and Loader, 2004; Kern et al., 2013). Consequently, the biochemical pathways supporting cellulose synthesis change during the growing season, where principally heterotrophic metabolism becomes autotrophic, thereby having an impact on the concentration of non-structural carbohydrate (Gilson et al., 2014), cellulose and lipid synthesis (Sessions, 2006; Tipple et al., 2013) among others.

In this study, we investigated the leaves and needles of long living deciduous- and evergreen species, including two oak trees (*Quercus robur* L.), a beech (*Fagus sylvatica* L.) and a yew tree (*Taxus baccata* L.). We assumed that carbon, oxygen, and hydrogen follow a similar isotopic seasonality in the foliage as have been documented in the xylem. The leaves were collected sequentially, through a complete growing season between April and October in 2012. Besides the alpha-cellulose yield, the stable isotopic composition of carbon, oxygen, and hydrogen was simultaneously measured in extracted alpha-cellulose (Loader et al., 2014) for the characterization of leaf development and intra-annual changes of cellulose synthesis at the leaf level.

## Materials and methods

### Site description

The sampling site is located in Bern, Switzerland and lies within the temperate climate zone, having a humid continental climate with warm summer (“Cfb” in Köppen-Geiger climate classification; Kottek et al., 2006). Climate norms for the 1981–2010 period, measured at the nearest meteorological station (Bern-Zollikofen; 46.9286°N, 7.4313°E, 553 m a.s.l.), were as follows: an average annual air temperature of +8.8°C, the mean temperature of the hottest month (July), +18.3°C, and for the coldest (January), −0.4°C. Due to the effect of its being located Alpine region, the annual precipitation is 1059 mm and August is the wettest month (116 mm), while February is the driest (55 mm) (Seiz and Foppa, 2011).

**Figure 1:**
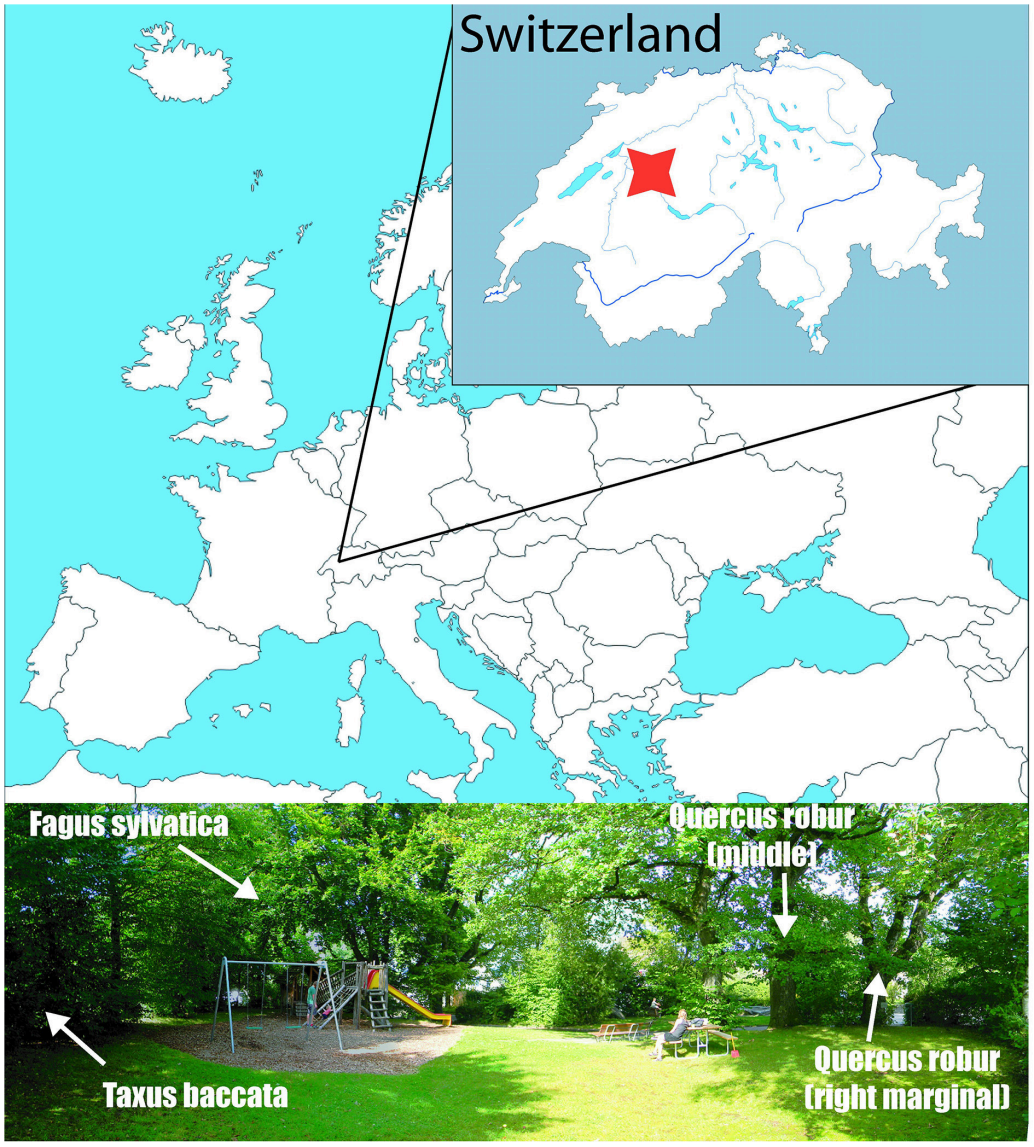
**Sampling site in Bern, Switzerland, denoted by the red mark (46.9283°N, 7.4317°E)**. The bottom picture shows the position of the sampled trees.

The sampling site is situated at the margin of the rural area (46.9283°N, 7.4317°E, 609 m) at the NE slope of Gurten Hill, on the outskirt of the City of Bern (Figure 1). The trees are mature specimens, growing next to each other and lacking any dominance effects. According to the Swiss regional phenological phases, the unfolding occurs at end of April (Rutishauser et al., 2007), similar to in central Europe (Michelot et al., 2012). Our observed unfolding date for the oak was a couple of days before, i.e., 116 Day Of Year (DOY), while for the beech and yew it happened between 116 and 122 DOY in 2012.

### Sampling of plant material

Two deciduous and one evergreen species, namely two pedunculate oaks (*Quercus robur* L.), a European beech (*Fagus sylvatica* L.), and a European yew (*Taxus baccata* L.) were sampled. The current foliar samples were harvested weekly in the first 3 weeks of the leaf development and with 11–21 day intervals through the rest of the growing season, depending on site accessibility, until leaf fall in October. The sampling covers the time period 116–281 DOY for the oaks, and 122–281 DOY for the beech and yew. The leaves were harvested from the same branches (at the same height and from the same side of the canopy) consistently to minimize sampling noise due to within-canopy isotopic variability. Two oak trees were sampled regularly to provide a verification test for the representativeness and reproducibility of the isotopic signature yielded by the sampling strategy. Regarding the sequentially harvested branch of the oaks, the right marginal oak tree was situated in a much more shaded sector of the canopy, while the branch of the middle tree bowed over a more open area, and hence represented a relatively more sun-exposed canopy sector. The samples were dried at room temperature, subsequently the broadleaves were separated for vein and blade. The “vein” phase contained the petiole, mid rib, and major veins.

### Cellulose extraction

Foliar samples were finely cut, and this was followed by measurements of the dry weight (DW) of the vein and blade samples before any chemical preparations were used, and afterwards alpha-cellulose was extracted and dried by the modified Jayme-Wise method (Boettger et al., 2007).

The alpha-cellulose yield results represent the ratio of the mass of DW to the mass of extracted alpha-cellulose yield, and are given in %.

(1) $$\begin{array}{r} \text{alpha cellulose yield }\left[\text{\%}\right]=\frac{{\text{m}}_{\text{alpha cellulose yield}}}{{\text{m}}_{\text{dry weight}}}\times \;100 \end{array}$$

We should note that, depending on the grain size of fine cut leaf/needle material, some part of it will stick in the pores of the filter during extraction. Therefore, we use the expression “yield” rather than “content” of cellulose.

### Sample preparation and isotope analysis of leaf alpha-cellulose

Overall, separated leaves of two deciduous and complete needles of an evergreen tree species were analyzed. The samples were homogenized to decrease possible uncertainties (Laumer et al., 2009), subsequently oven-dried (50°C), weighed, and placed in silver capsules.

For triple-isotope analysis (Loader et al., 2014), we used conventional Isotope Ratio Mass Spectrometry coupled to a Pyrolyser (HEKAtech GmbH, Germany), which is similar to the previously used TC/EA the technical details are described by Leuenberger (2007). However, this approach is extended to measurements of non-exchangeable hydrogen of alpha-cellulose using the on-line equilibration method (Filot et al., 2006). Results are reported in permil (‰) relative to the Vienna Pee Dee Belemnite (VPDB) for carbon and to Vienna Standard Mean Ocean Water (VSMOW) for hydrogen and oxygen (Coplen, 1994), using the traditional “δ” notation

(2) $$\begin{array}{r} \delta \text{X}\left[‰\right]=\left(\frac{{\text{R}}_{\text{Sample}}-{\text{R}}_{\text{Standard}}}{{\text{R}}_{\text{Standard}}}\right)\cdot 1000 \end{array}$$

where X stands for D, ^13^C, ^18^O, R stands for ^2^H/^1^H, ^13^C/^12^C, or ^18^O/^16^O in a sample and standard, correspondingly.

Samples were measured in triplicates and their standard deviations were 90% to within 0.2‰ for carbon, 93% to within 3‰ for hydrogen and 90% to within 0.3‰ for oxygen isotopes with overall mean standard deviations of 0.12, 1.83, and 0.28‰ for carbon, hydrogen and oxygen, respectively.

### Meteorological data

**Figure 2:**
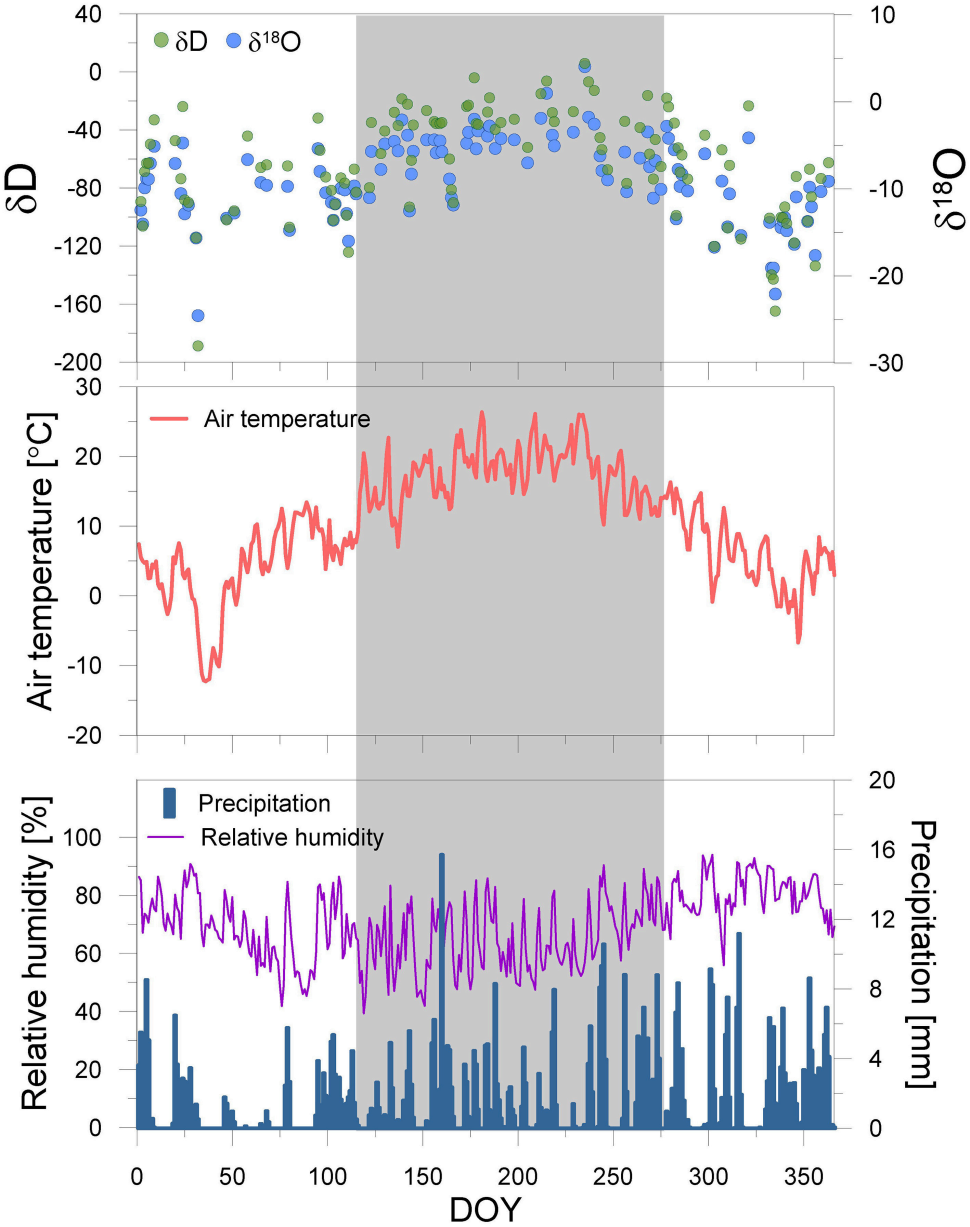
**Seasonal course of the meteorological parameters temperature, precipitation and relative humidity, and the water stable isotopes of precipitation at the sampling site in 2012**. The date is reported in Day of Year (DOY). The deep blue bars, purple- and red line, green- and light blue circles represent the precipitation [mm], relative humidity [%], temperature [°C], and δD and δ^18^O of precipitation [‰], respectively. The gray shaded area denotes the sample period for leaves.

Meteorological datasets are supported by the STARTWAVE atmospheric water monitoring system (Morland et al., 2006), providing a homogenized high resolution (10 min) air temperature and relative humidity dataset in Bern. However, we measured daily precipitation events and their hydrogen and oxygen isotopic composition in 2012. The δD and δ^18^O of precipitation are expressed in permil (‰) relative to the Vienna Standard Mean Ocean Water (VSMOW) for hydrogen and oxygen (Figure 2).

### Mechanistic modeling

For a better understanding of the seasonal leaf expansion (leaf cellulose), we used the Péclet-modified Craig-Gordon Model (PMCG) (Kahmen et al., 2011) to calculate hydrogen and oxygen isotopic composition of leaf water taking into consideration results from related other studies (Dongmann et al., 1974; Farquhar and Lloyd, 1993; Roden and Ehleringer, 2000).

(3) $$\begin{array}{l} \delta {\text{D}}_{\text{LC}}={\text{p}}_{\text{ex}}\times {\text{p}}_{\text{x}}\times (\delta {\text{D}}_{\text{SW}}+\varepsilon_{\text{HH}})\\ \;+\;(1-\;{\text{p}}_{\text{ex}}{\text{p}}_{\text{x}})\times (\delta {\text{D}}_{\text{LW}}+\varepsilon_{\text{HA}}) \end{array}$$

(4) $$\begin{array}{l} \delta^{18}{\text{O}}_{\text{LC}}=\;{\text{p}}_{\text{ex}}\times {\text{p}}_{\text{x}}\times (\delta^{18}{\text{O}}_{\text{SW}}+\varepsilon_{\text{OH}})\\ \;+\;(1-\;{\text{p}}_{\text{ex}}{\text{p}}_{\text{x}})\times (\delta^{18}{\text{O}}_{\text{LW}}+\varepsilon_{\text{OA}}) \end{array}$$

where δD_LC_ and δ^18^O_LC_ represent the hydrogen and oxygen isotopic composition of leaf cellulose, p_ex_ represents the proportion of exchangeable oxygen in cellulose formed from soluble carbohydrates, p_x_ represents the proportion of source water, δD_SW_ and δ^18^O_SW_ represent the isotopic composition of source water, ε_HH_/ε_OH_ and ε_HA_/ε_OA_ represent the overall heterotrophic and autotrophic fractionation between hydrogen and oxygen atoms of source water and photosynthate, respectively. Finally, δD_LW_ and δ^18^O_LW_ represent the isotopic composition of leaf water.

Consequently, the hydrogen and oxygen isotopic composition of leaf water (δD_LW_, δ^18^O_LW_) can be determined as

(5) $$\begin{array}{r} \delta {\text{D}}_{\text{LW}}\;=\;\frac{\delta {\text{D}}_{\text{LC}}\;-\;{\text{p}}_{\text{ex}}{\text{p}}_{\text{x}}\left(\delta {\text{D}}_{\text{SW}}\;+\;\varepsilon_{\text{HH}}\right)}{\left(1-{\text{p}}_{\text{ex}}{\text{p}}_{\text{x}}\right)}\;-\;\varepsilon_{HA} \end{array}$$

(6) $$\begin{array}{r} \delta^{18}{\text{O}}_{\text{LW}}\;=\;\frac{\delta^{18}{\text{O}}_{\text{LC}}\;-\;{\text{p}}_{\text{ex}}{\text{p}}_{\text{x}}\left(\delta^{18}{\text{O}}_{\text{SW}}\;+\;\varepsilon_{\text{OH}}\right)}{\left(1-{\text{p}}_{\text{ex}}{\text{p}}_{\text{x}}\right)}\;-\;\varepsilon_{OA} \end{array}$$

The leaf water isotope values were calculated using current measurements of leaf cellulose δD_LC_ and δ^18^O_LC_, 0.4 for p_ex_, 0.65 for p_x_ after Sternberg and Cernusak (Cernusak et al., 2005; Sternberg, 2009), 21.7‰ for ε_OH_ (Guy et al., 1993), and 27‰ for ε_*OA*_ (Epstein et al., 1977; Deniro and Epstein, 1981; Sternberg and Deniro, 1983). In addition, during water uptake no isotope fractionation occurs (White et al., 1985), therefore we defined p_x_ as 1 regarding the isotopic composition of vein water. This assumption is equivalent to the vein leaf water value being equal to the source water in the case of the absence of a back diffusion effect of enriched leaf water from blades into veins. ε_HH_ and ε_HA_ are calculated as +158 and −171‰, respectively (Yakir and Deniro, 1990). For the isotopic composition of source water, we assumed homogenized δD and δ^18^O of site precipitation as indicated by the observed low fractionation in the soil at a different site (Treydte et al., 2014). The aforementioned mechanistic models were applied to all species and leaf compartments to determine the δD_LW_ and δ^18^O_LW_.

### Statistics

The *t*-tests of independent-samples were conducted on unrelated groups of tree species or leaf compartments (vein vs. blade) using cellulose yield and isotope values as parameters to compare their group means objectively. The difference was taken to be significant for *p* < 0.05. STATISTICA (StatSoft Inc., 2011) was used for calculations.

## Results

### Alpha-cellulose yield of DW

**Figure 3:**
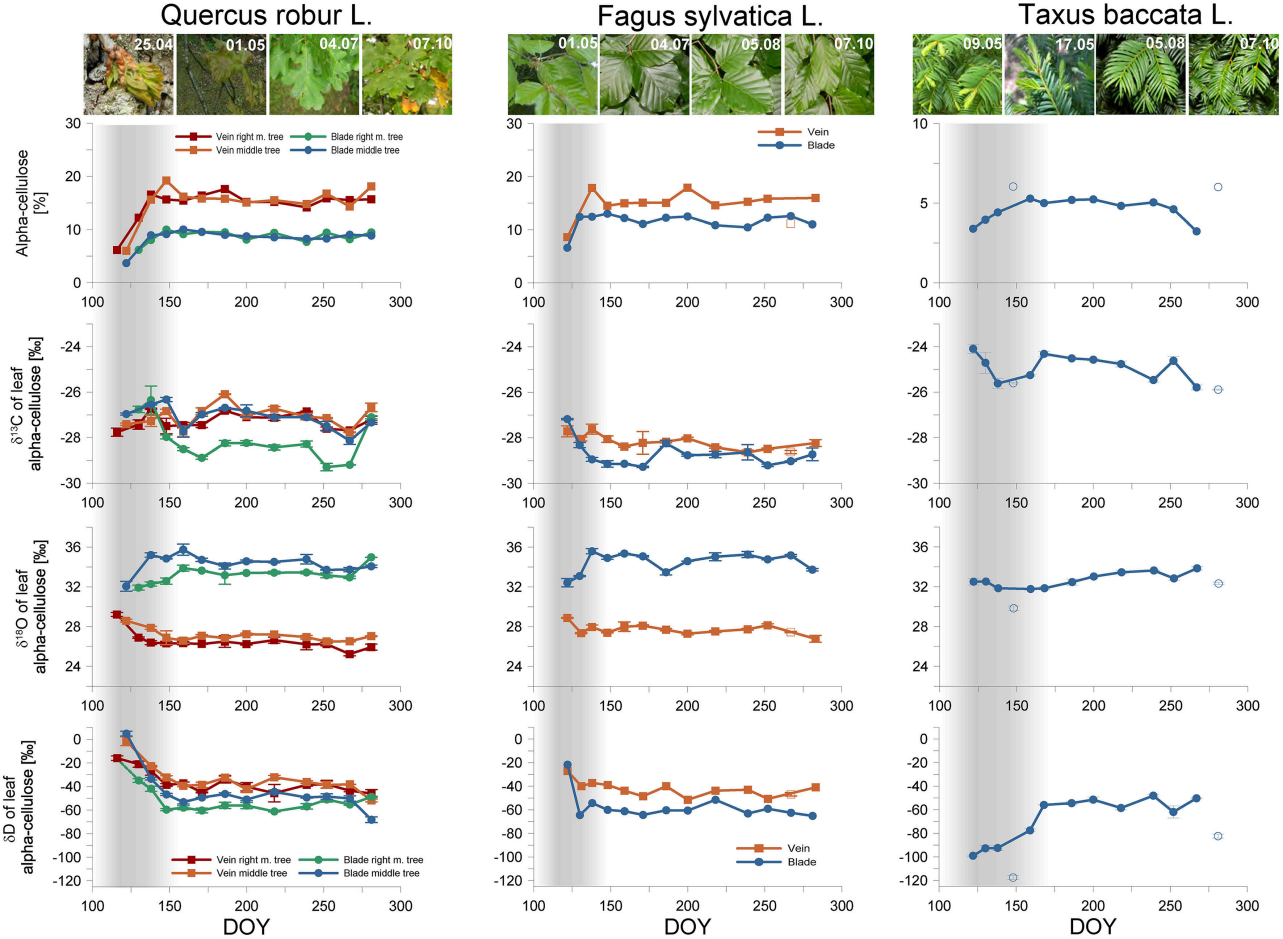
**Alpha-cellulose yield [%], carbon-, hydrogen- and oxygen isotopic composition [‰] of oak (***Quercus robur L***.), beech (***Fagus sylvatica L***.), and yew (***Taxus baccata L***.) foliage**. The x-axis represents the Day of Year (DOY) in 2012. For oaks, the red, green, orange, and blue lines represent the veins and blades of the right marginal tree, and the veins and blades of the middle tree, respectively. For the beech, the orange and blue lines represent the veins and blades, respectively. The gray shaded area denotes the juvenile period for all species. Note that squares and circles denote veins and blades, respectively. Open symbols mark values corresponding to potential cellulose extraction failures.

Similar foliar cellulose yields were observed in the case of the oaks and beech (Figure 3). Yew needles had a significantly lower alpha-cellulose yield than deciduous species (*p* < 10^−8^ and *p* < 10^−7^ in any comparison for full and extraction failure excluded datasets, respectively), which may be plausibly explained by the distinct anatomical structures (i.e., lower amounts of xylem) of needles compared to broadleaves (Spjut, 2007).

Anomalous cellulose yields indicated some extraction failure in three cases (see Figure 3). A vein sample for beech yielded an unusually low amount of cellulose, indicating sample loss, probably due to the opening of the filter bag. However, all of the derived isotope ratios fitted nicely into the seasonal trends, and therefore the isotopic values were kept. Two yew needles (the fourth and the last) yielded a higher amount of cellulose than the rest of the samples. Although the corresponding δ^13^C-values fit nicely into the seasonal trend, the water isotopes clearly depart from the inter-annual trend. It may be supposed that further depleted residuum remained in the filter bag besides the alpha-cellulose with regard to its oxygen and hydrogen composition. These values were omitted from the discussion.

As may be logically expected, the lowest amount of alpha cellulose was found in the leaf samples taken at the initiation of the leaf development. Moreover, at this time the blades and veins of oak and beech similarly started from 5 to 7%. In the course of the following days, the characteristics of the blades and veins evolved, and simultaneously the alpha-cellulose yield increased, until the signals became continuously consistent for blades (10–12%), and also for veins (16%). Based on these characteristically distinct patterns, seasonal leaf development can be divided into a juvenile and a mature phenophase. Increasing cellulose yields indicate that foliage was still in the expansion phase (juvenile period) while the stabilized cellulose yields suggest that the foliage is complete (mature phase). The juvenile phase was shortest (<16 days) for beech, evidencing more dynamic blade expansion than it is for oak (Gond et al., 1999; Bequet et al., 2011), while a twice-as-long juvenile phase was observed for oak and yew (>30 days).

More specifically, for oaks, after the mature phase had begun, the cellulose content did not change, inducing a steady offset with a ~7% higher cellulose yield for veins. On the contrary, for beech, the alpha-cellulose yield of blades and veins frequently fluctuated between 1.5 and 5%, with an average offset of ~4% during the mature phase. For yew, the cellulose yield signal is weaker during the juvenile phase period. It starts from ~3% and became steady at around the 5% level representing a balanced leaf expansion.

In addition, the juvenile and mature phases of foliar development were accompanied by characteristic changes in the stable isotope composition of leaf cellulose.

### The carbon isotopic composition of leaf alpha-cellulose

We documented the expected seasonality only for blades and needles, starting with more enriched values and decreasing afterwards, reflecting the previously documented seasonal carbon isotope pattern in tree rings (Damesin et al., 1998; Helle and Schleser, 2004). The veins of oaks and beech show no clear difference in δ^13^C during the growing season (Figure 3). However, the carbon isotopic composition of oak leaves had a slightly more positive mean during the mature phase (δ^13^C_oak−blade_ = −27.84 ± 0.86‰, δ^13^C_oak−vein_ = −27.10 ± 0.42‰) compared to beech leaves (δ^13^C_beech−blade_ = −28.91 ± 0.34‰, δ^13^C_beech−vein_ = −28.33 ± 0.22‰). Nevertheless, yew needles presented more positive values than either deciduous species, with an annual mean of δ^13^C_yew_ = −24.96 ± 0.54‰ (Figure 3).

### The oxygen isotopic composition of leaf alpha-cellulose

Oxygen isotope measurements show a distinct individual characterization of the foliage type (broadleaf vs. needle), and for structural parts (vein vs. blade) as well. Veins are less enriched compared to blades, starting from 29‰ and decreasing to 27‰ for oaks and starting from 29‰ and decreasing to 28‰ for beech during the juvenile phase (Figure 3). In the mature phase, the δ^18^O veins of both tree species were practically constant. Blade cellulose started from 32‰ in both cases, increasing during the juvenile phase up to 36‰, remaining constant during the mature phase. The yew represented an annual mean value of δ^18^O_yew_ = 33.21 ± 0.52‰, with no visible sign of a juvenile-mature difference (Figure 3). However, the mean values of the mature phase are δ^18^O_oak−vein_ = 26.57 ± 0.57 ‰, δ^18^O_oak−blade_ = 33.89 ± 0.65‰ for the oaks, and δ^18^O_beech−vein_= 27.61 ± 0.42‰, δ^18^O_beech−blade_ = 34.85 ± 0.57‰ for the beech.

### The hydrogen isotopic composition of leaf alpha-cellulose

The seasonal variability found for hydrogen isotopes is not similar to that of carbon and oxygen isotopes.

Since the blades and veins follow a distinct signal in the cases of carbon and oxygen, in the case of hydrogen the only difference observed was the higher enrichment of veins compared to blades for deciduous species (Figure 3). The first oak leaf samples started with highly enriched values and decreased during the juvenile phase. The mean values are δD_oak−blade_ = −53.34 ± 6.26‰, δD_oak−vein_ = −39.94 ± 5.24‰ during the mature phase. However, the offset between the first sampling and the mean mature phase values varies between 18 and 57‰. For beech, a very similar seasonal pattern occurred, but due to the shorter juvenile phase the offset was less resolved. The annual dataset begins from −20‰ for vein and blade and decreases to −40‰ for vein and −60‰ for blade, in a similar way to the case of the oaks, described above. The mean values during the mature phase are δD_beech−blade_ = −60.31 ± 3.69‰, δD_beech−vein_ = −38.75 ± 4.43‰, resulting in an offset of 20–40‰.

The yew presented a different intra-annual hydrogen isotopic composition change (Figure 3). During the juvenile phase, the lowest δD-values were observed between −100 and -90‰ which subsequently increased during the mature phase to δD_yew_ = −54.38 ± 4.86‰, resulting in an offset of 44.65‰.

### The results of leaf water simulation

**Figure 4:**
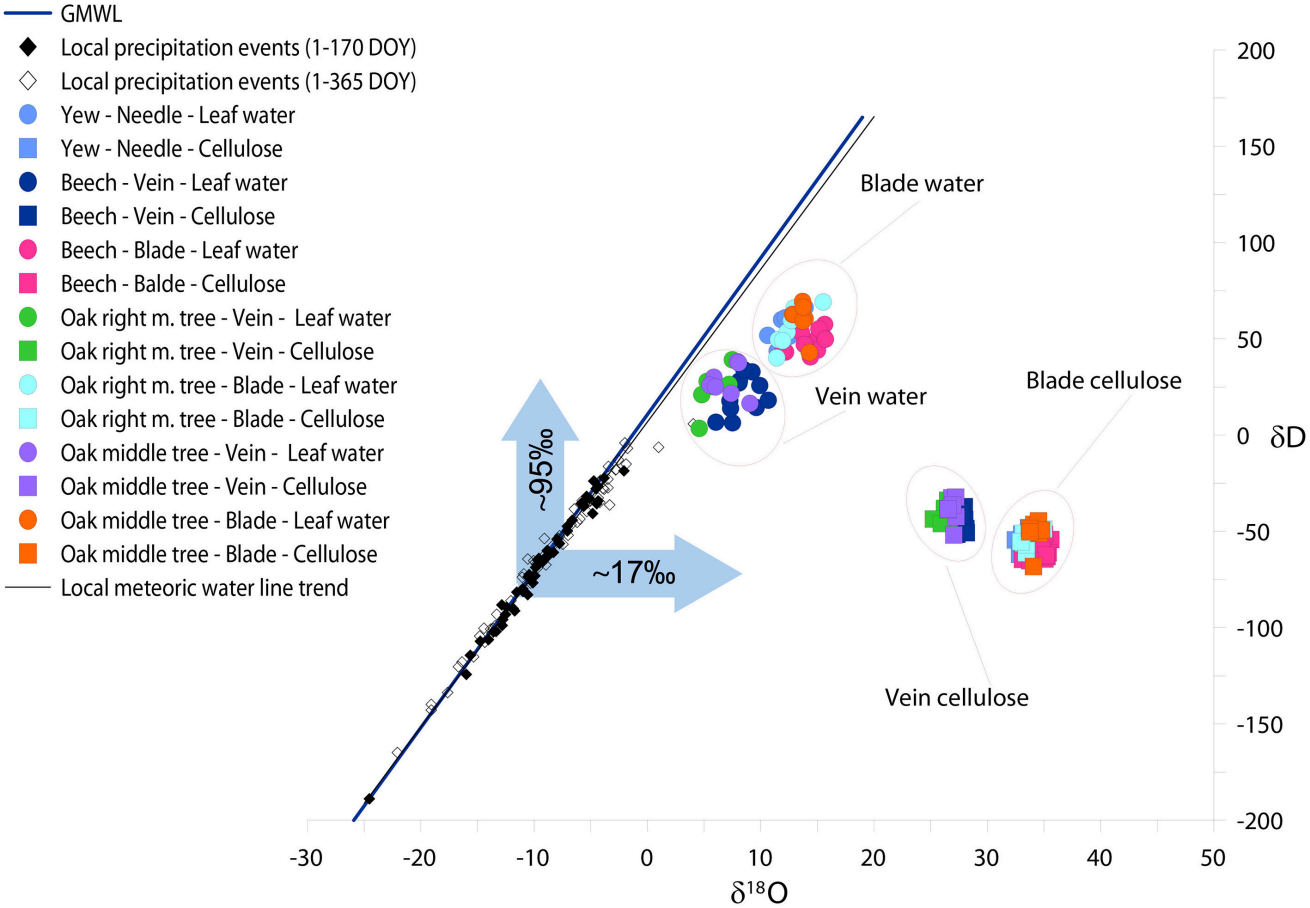
**The relationship between δD and δ^18^O of local precipitation, species specific calculated leaf water, and measured leaf cellulose in mature leaves (DOY 150–365)**. Different colors denote different trees. Circles, squares, open, and closed diamonds denote the isotopic composition of leaf water, cellulose, precipitation events during 1–365 DOY and precipitation events during 1–170 DOY, respectively. The blue arrows denote the isotopic shift of δD and δ^18^O of vein leaf water compared to the annual mean δD and δ^18^O of precipitation. The blue and black lines represent the global and local meteoric water line, respectively.

Applying the Péclet-modified Craig-Gordon Model (PMCG) we estimated hydrogen and oxygen isotopic composition of leaf water regarding veins, blades and needles (Figure 4). Since, we defined p_x_ as 1 in order not to perform fractionation during water transport from the roots to the leaf level, the δD and δ^18^O of veins became less positive than blades. δ^18^O_vein_ of deciduous species varies between 5.8 and 8.37‰ (δ^18^O_vein−oakmid_ = 7.10‰, δ^18^O_vein−oakright_ = 5.84‰ and δ^18^O_vein_-_beech_ = 8.37‰) while the δ^18^O_blade_varies in the range from 12.61 to 14.29‰. Yew, similarly as oak and beech blades, represents 12.05‰ for δ^18^O of needle.

We found an offset between veins and blades but similar trends for hydrogen isotopes. δD of oak and beech veins are less positive (δD_vein−oakmid_ = 27.84‰, δD_vein−oakright_ = 24.53‰ and δD_vein−beech_ = 20.45‰) than blades (δD_blade−oakmid_ = 60.52‰, δD_blade−oakright_ = 55.27‰ and δD_blade−beech_ = 48.39‰). As it was found for oxygen isotopes, yew fits to deciduous species, namely δD_yew_ = 51.09‰.

## Discussion

The stable carbon, oxygen, and hydrogen isotope composition of the alpha-cellulose of foliage of the three species was monitored throughout the growing season in 2012 (from 115 DOY until 281 DOY). The investigated trees are growing next to each other, and therefore the atmospheric (weather, ambient CO_2_, etc.) and soil moisture conditions were identical for all trees. Consequently, we can compare the leaf cellulose formation of the studied species by investigating the isotopic composition of all elements (C–O–H) of cellulose molecules. Besides, tree ring anatomy and the pathways of metabolic processes also change during growing season (Hayes, 2001). At the very beginning of the growing season, when the tree is not yet ready for CO_2_ fixation, remobilized carbohydrates dominate the sources. These reserves are converted into metabolic intermediates and applied to proteins, carbohydrates, lipids, etc. through biosynthesis, providing indirect sources of carbohydrates from previous years. During the juvenile phase, similarly to the EW phase in tree rings, the dominance of indirect carbohydrate sources decreases besides the increase of products formed by photosynthesis. Despite depending on climate conditions during summer, carbohydrates can be formed by either heterotrophic metabolism under non-optimal or by autotrophic metabolism under optimal conditions (Kimak and Leuenberger, 2015), new assimilates control the isotopic signal in tree-ring cellulose. In addition, depending on whether the tree utilizes carbohydrates directly or indirectly, the product serves as an input of downstream processes fingerprinted in the stable isotopic composition of tree ring cellulose (Kagawa et al., 2006).

### Coherency between individual trees

The sampled oak trees presented a strikingly similar seasonal course regarding cellulose yield and water isotope signals. The carbon isotope fluctuations presented are also similar, although the mature blade of the right marginal oak tree (Figure 3 green line) had an offset (1–1.5‰) compared to other blades or veins. This offset can plausibly be explained by a shaded canopy effect with decreased light conditions (see Section Sampling of the Plant Material) since decreasing irradiance in the canopy generates more negative δ^13^C-values in the foliage (Hanba et al., 1997) due to the decreased assimilation rate and consequently the increased c_*i*_/c_*a*_ ratio relative to the stomatal conductance (Jones, 1998). Note, that the offset is not presented for the first and the last leaf samples therefore we cannot exclude other influences that could explain this variable offset.

The strong coherency found between the sampled trees verifies the sampling strategy and confirms that representative isotopic signals can be observed allowing a physiological interpretation of the plants.

### Quercus robur

The alpha-cellulose yield of the leaf dry weight shows well-expressed differences between phenological phases, clarifying the switch from the juvenile to mature phases of leaves (Figure 3). The juvenile phase started with a lower alpha-cellulose content and increased until the leaf area maximizes along with the maturing of the leaves. The juvenile-mature turning point came at ~150 DOY, consequently the juvenile phase lasted ~35 days. In addition, the alpha-cellulose yield of veins and blades indicates that veins are more readily comparable with the alpha-cellulose yield of tree rings than blades, although the observed veins contain less alpha-cellulose than tree rings (Rowell, 2012). However, the steady offset between veins and blades manifest in progressive leaf development after budburst.

Although the expected seasonality of δ^13^C is not clearly represented in the case of oaks, the values of δ^13^C show a good agreement with previous investigation documenting δ^13^C-values in the range from 25.5 to 28‰ regarding oak bulk leaf material (Eglin et al., 2009; Maunoury-Danger et al., 2012) having an inter-annual variability of 2.5‰ that was also documented for other species living under different environmental condition (Ehleringer et al., 1992; Wang and Schjoerring, 2012).

Veins for both oak trees indicate positive peaks during the juvenile phase, and a decreasing trend until the mature phase. However, on 159 DOY, notably less enriched values occurred that might be an influence of stomatal conductance, consequently on the ratio of c_*i*_/c_*a*_ and discrimination against heavy isotopes by Rubisco, indicated by considerable amounts of precipitation between 150 DOY and 175 DOY (Figures 2, 3). In addition, this observation was also made in the case of the beach leaves and the yew too. On the contrary, the oxygen isotopic composition of veins and blades showed remarkable differences over the growing season, indicating either (i) alternative metabolic processes, or (ii) alternative sources for oxygen isotopes. Since the source water is directly transported from the soil through the roots and stem to the branches without any fractionation (White et al., 1985), the responsible fractionation are probably the biochemical processes acting at leaf level. The backward projected juvenile trends of vein and blade δ^18^O cellulose point to a shared source water with a ~30.5‰ oxygen isotope composition. Over time the δ^18^O_blade_ presented an increasing trend due to leaf formation related to higher evaporative enrichment, until it is maximized by the end of the juvenile phase, and remained consistent during mature phase. Consequently, the difference between the usage of indirectly and directly synthetized carbohydrates is approximately 3‰. Contrary, δ^18^O_vein_ showed a moderately decreasing trend during the juvenile phase that we assign to the diminishing role of more enriched soluble carbohydrates and sap water stored over winter that is mixed and is subsequently dominated by source water. However, if we assume the first point was formed from soluble carbohydrates and old sap water and δ^18^O_LW_ is formed from source water we can establish the difference (at ~1–2‰).

The seasonality of the hydrogen isotopic composition of oak leaf cellulose showed individual trends compared to the previously discussed isotopes. In particular, the δD of veins and blades represented more enriched values induced by indirectly produced carbohydrates and decreased until the end of the juvenile period. These patterns provide clear evidence for the transition between different metabolic pathways. However, due to the higher exchange rate of hydrogen within biochemical processes (Augusti et al., 2006), no significant difference was found for the δD of veins and blades of the particular trees, namely ~0‰ for the middle tree and approximately –20‰ for the right marginal tree. Despite this, we found strong correlations between the veins and blades of the right marginal tree (*r*^2^ = 0.82) and the middle tree (*r*^2^ = 0.96), the blades are slightly shifted toward more depleted compositions during mature phase. The offset between blades and veins is 16.75‰ for the right marginal tree, 11.81‰ for the middle tree.

However, the difference between indirectly and directly formed carbohydrates is 22, 24, 53, and 21‰ for the blades and veins of the right marginal tree, blades, and veins of the middle tree, respectively. These differences are supported by δD of leaf wax n-alkanes (nC-27 and nC-29) measurements (Newberry et al., 2015).

### Fagus sylvatica

Although, oak and beech are both deciduous, many differences occur in their tree ring anatomy. The beech xylem ring includes many vessels scattered over the entire year, therefore the visual determination of juvenile and mature phases is more ambiguous (Čufar et al., 2008). However, our observations verify the existing characteristics of intra-annual differences in phenological phases. Alpha-cellulose yield results document faster leaf expansion and consequently, a shorter leaf growing period (<16 days) for beech than for oaks and yew. The veins, similar to those of oaks, contain a higher proportion of alpha-cellulose than blades, but the alpha-cellulose content of the blades is closer to that of veins, and dissimilar to that of oak, resulting in a mean offset of ~4% during mature phase (Figure 3).

Despite beech leaf cells being formed faster than those of oaks, we found a clear seasonality in intra-annual isotope measurements. The carbon isotopic composition of leaf alpha-cellulose confirms the aforementioned faster leaf cellulose development. Thus, a decreasing trend occurred during the juvenile phase, starting with more enriched δ^13^C until the leaf area and therefore, the assimilation rate is maximized. In addition, veins did not mirror the blades' variability. We assign this to the higher usage of reserved carbohydrates.

The δ^18^O of leaf compartments showed similar results to those of the oaks (Figure 3). Since blades are supposed to follow the gradual increase of evaporative enrichment at leaf level, it displays a positive trend through the juvenile period. However, we found lower seasonal variation for veins, which might indicate a faster consumption of the remaining soluble carbohydrates and sap water from winter and the earlier usage of soil water.

The hydrogen isotopic composition of veins and blades demonstrates practically the same seasonal variability as found in oaks. The observed fractionation between indirect and direct cellulose synthesis can be estimated as 17.4‰ for veins and 38.6‰ for blades.

### Taxus baccata

As the single evergreen in our study, the yew has a species-specific annual xylem ring anatomy (Vaganov et al., 2009) and isotopic composition (Barbour et al., 2002). Despite, intra-annual changes in δ^13^C of the tree ring cellulose, typically reflecting environmental conditions (Leavitt and Long, 1991), the influence of previous years' storage on current year xylem formation is also well-documented (Schubert and Jahren, 2011). The needles of *Taxus baccata* include more mesophyll than vascular tissue (Schirone et al., 2010), therefore these results should be regarded as a blade-dominated mixture of blade and vein signals. This has to be taken into consideration when a comparison is to be made with the broadleaf patterns.

We found a lower percentage of alpha-cellulose yield for yew than for deciduous species, and the seasonal variability also decreased. In particular, needle formation was accompanied by a moderated increase from 3 to 5% during the juvenile phase, and remained constant up to the end of the mature phase.

Surprisingly, the δ^13^C of needles shows similar activity for the yew as compared to the oaks and beech, namely the impact of previous years' carbohydrates fingerprinted in juvenile phase tissues, indicating a negative tendency at the start of growing period. Moreover, the less enriched δ^13^C-values after 150 DOY, documented for all species, might be the aforementioned collective impact of heavy precipitation events between 150 and 175 DOY (Figure 3). In addition, yew needles have significantly (*p* < 10^−14^ in any combination) more enriched values (3–5‰) than deciduous species.

Due to the lack of leaf tissue separation for yew, the oxygen isotopic composition represents also a bulk of veins and blades, including values closer to that of the δ^18^O of deciduous species. It might be reasoned that the domination of blade tissues within needles is responsible for this finding.

In contrast to the oaks and beech, the yew has a particular seasonality in the case of hydrogen isotopes (Figure 3). Due to the presence of needles from previous years, the variability of indirect and direct cellulose synthesis disappears and the direct assimilates became the major driver for cellulose formation during the juvenile phase, too. Therefore, the δD-values of leaf cellulose represent the current water conditions, consequently the δD of precipitation. These results are in agreement with the findings of Treydte et al. (2014) namely, that the oxygen isotopic composition of tree ring cellulose predominantly mirrors the seasonal trends of the source water.

### The characterization of leaf compartments based on δD and δ^18^O measurements and model results

Since leaf cellulose synthesis, in common with tree-ring cellulose synthesis, is supported by the reserves from previous years during the leaf expansion stage, the carbon isotopic composition of leaf cellulose is also influenced by stored carbohydrate, affecting or even masking the expected seasonal variations at the leaf level. On the contrary, the stored carbohydrates have much less influence on hydrogen and oxygen isotopes, due to their exchangeability. There is no exchange during cellulose synthesis in the positions of certain oxygen atoms (Waterhouse et al., 2013), thus decreasing the impact which might be expected of carbohydrate storage on cellulose formation during the juvenile phase.

The exchangeability of hydrogen isotopes in cellulose varies widely. Generally, the higher the isotope exchange rate for specific molecule positions is, the more representative the isotope composition of that particular position for physiological processes is. While in the opposite case, specific molecule positions are influenced primarily by source water, and so the actual climate conditions (Augusti et al., 2008). Because in alpha-cellulose the majority of hydrogen positions with strong isotope exchange rates are represented, our results of bulk veins and blades, we expect that they should reflect mainly the seasonal changes in metabolic processes.

The water isotopes in vein and blade cellulose clearly indicate temporal and spatial patterns, including (i) the seasonal changes of the autotrophic and heterotrophic metabolisms, and (ii) distinction of cellulose synthesis between leaf tissues. For species where the separation of blades and veins was available, the oxygen and hydrogen signal is clearly different at the beginning of leaf formation driven by autotrophic metabolism compared to the mature phase, where both oxygen and hydrogen fractionations are dominated by heterotrophic metabolism (Figure 3). During the leaf expansion phase, the evaporative enrichment and the Péclet-effect exercise an increasing effect on oxygen isotopes, until the leaves are fully developed, and the aforementioned processes are maximized for blades, while these impacts are less effective for veins.

As with oxygen, hydrogen isotopes also represent the heterotrophic metabolism at the beginning of leaf formation and become less enriched due to the accelerated exchange of NADP^+^-NADPH reactions during photosynthesis. However, the hydrogen results of blades and veins share the same seasonality but with more enriched isotopic composition characterizes the veins (15–20‰).

As may be seen in Figure 4, a discrepancy was found between the isotopic signal of vein water and the measured isotopic ratio of site precipitation (source water), where hydrogen and oxygen isotopic compositions are more enriched. Due to the fact that water uptake is not accompanied by isotope fractionation, we attribute this to a mixing effect, particularly as vein cellulose is formed not only by the source water but by a mixture of non-enriched soil water and evaporative enriched laminar water originating from the photosynthetic active part of the leaf. These effects result in an approximately +95‰ enrichment for hydrogen and +17‰ for oxygen for modeled isotopic composition of vein water compared to local precipitation events.

## Conclusions

We investigated the stable carbon, oxygen, and hydrogen isotope signatures of the leaf samples of two deciduous and one evergreen species during the growing season in 2012. Although, the deciduous species have good coherences, the characterization by the stable isotope approach revealed species-specific variations between phenology phases and activities. Yew has the lowest and beech the highest degree of leaf expansion activity. However, after the leaves are complete (mature phase), the values represent the foliage variability of the sampling site, exhibiting very good coherences.

Separated leaf tissues combined with the triple isotope approach allow us to document seasonality in leaf development driven by carbohydrate sources from previous years on the one hand, and by the alternation of heterotrophic and autotrophic metabolism on the other. In addition, due to the different utilization of carbon, oxygen, and hydrogen isotopes, alternative metabolic processes produce individual seasonality for each isotope for the studied species.

## Author contributions

AK performed the stable isotope analyses and drafted the first version of the manuscript. ZK conceived the study and collected the samples. ML contributed to the evaluation of the results. All authors provided comments to improve the manuscript.

## Funding

AK and ML acknowledge the financial support for the Swiss National Science Foundation (SNF-iTREE, project number: 136295). ZK and ML thanks the Scientific Exchange Programme between Switzerland and Hungary (project code: 10.255) for the financial support. ZK acknowledges the support for the “Lendület” program of the Hungarian Academy of Sciences (LP2012-27/2012).

### Conflict of interest statement

The authors declare that the research was conducted in the absence of any commercial or financial relationships that could be construed as a potential conflict of interest.
